# Isolation and Structural Elucidation of Novel Antidiabetic Compounds from Leaves of *Momordica balsamina* Linn and *Leptadenia hastata* (Pers) Decne

**DOI:** 10.22037/ijpr.2020.113632.14440

**Published:** 2021

**Authors:** Nafisatu Kabir, Ismail A Umar, Habila A Dama, Dorcas B James, Hajiya M Inuwa

**Affiliations:** a *Department of Biochemistry, Faculty of Science, Federal University Dutse, Jigawa state-Nigeria. *; b *Department of Biochemisty, Faculty of Life Sciences, Ahmadu Bello University Zaria, Kaduna State-Nigeria. *; c *Department of Chemistry, Faculty of Physical Sciences, Ahmadu Bello University Zaria, Kaduna State-Nigeria.*

**Keywords:** Triterpenoids, Isoflavonoids, Antidiabetic, Mormordica balsamina, Lepatdenia hastata

## Abstract

The antihyperglycemic effect of the polyherbal combination of the leaves of *Momordica balsamina* Linn (MB) and *Leptadenia hastata* (pers) Decne (LH) have been reported in our previous study in addition to its documented dietary usages. However, the bioactive principles are yet to be fully elucidated. In the present study, bioactive antidiabetic compounds from the leaf extracts of *Momordica balsamina* Linn and *Leptadenia hastata* (pers) Decne were isolated and characterized. The plant leaves were fractionated with solvents in ascending order of polarity (hexane-chloroform-ethylacetate-methanol) using microwave assisted extraction method. The ethylacetate (MBE) and methanolic (LHM) leaf extracts of MB and LH, having the highest antihyperglycemic effects were purified by column chromatography and preparative thin layer chromatography. The antihyperglycemic activity of the isolated compounds was evaluated in streptozotocin (STZ)-induced diabetic rats and the structures of the most bioactive compounds were elucidated by ^1^H and ^13^C Nuclear Magnetic Resonance (NMR) spectroscopy in comparison with reported literature. A pentacyclic triterpenoid (H3) and an isoflavone (LH2b) isolated from MBE and LHM with significant (*p < *0.05) antihyperglycemic effects were identified as betulinic acid and 5-methyl genistein respectively. Our study isolated for the first time a triterpenoid and an isoflavone with potential antidiabetic effects from these indigenous antidiabetic plants. This further validates the traditional multi-therapeutic usage of the combination for the management of Diabetes Mellitus (DM) and its complications.

## Introduction

Diabetes mellitus is a complex metabolic disorder characterized by chronic hyperglycemia, hypo-insulinemia and ketosis. Persistent hyperglycemia of diabetes mellitus has been associated with cardiovascular diseases, retinopathy, neuropathy, nephropathy, and other long-term complications (1). In Africa more than 14 million people have diabetes mellitus, accounting for about 4.3% of adults with diabetes in the region (2). According to the World Health Organization (WHO), more than 1,200 plant species worldwide are used for the treatment of diabetes mellitus and a substantial number of the plants have shown effective hypoglycemic activity after laboratory testing (3). The vast majority of these plants contain varied amounts of different classes of phytochemical compounds such as glycosides, alkaloids, terpenoids, flavonoids, carotenoids and phenolic compounds that have frequently been documented to have antidiabetic effects (4). The primary goal of diabetes mellitus treatment is to achieve reasonable glycemic control to prevent the onset of long-term complications. Although several therapeutic agents have been developed for diabetes mellitus treatment, the main therapeutic goals are yet to be achieved entirely. Studies have shown that several indigenous herbal medicines appear to be effective for treating diabetes mellitus and its complications (5). Most traditional herbal healers use the indigenous antidiabetic plant parts as a polyherbal combination with the aim of achieving a multi-therapeutic antidiabetic effect. 

*Leptadenia hastata *(Pers) Decne and *Mormodica Balsamina* L.; commonly used for dietary purposes, are few of such indigenous medicinal plants traditionally reported to have diverse hypoglycemic activities in many countries especially in Asia, Latin America, and Africa (6). The leaves, fruits, seeds, and bark of *Mormordica balsamina *and *Leptadenia hastata* have been reported to contain resins, alkaloids, flavonoids, glycosides, steroids, terpenes, cardiac glycoside, tannins, proanthocyanidins, alkaloids and saponin with distinct pharmacological activities (6-8). Studies using laboratory animal models of diabetes showed that *Momordica *species help prevent or counteract Type II diabetes, improve glucose tolerance and suppress post-prandial hyperglycemia by inhibiting alpha glucosidase (9). Activity based reviews of *Momordica balsamina* indicated that it possess other biological activities like antimicrobial, antispasmodic, anti-inflammatory, analgesic, anti-HIV, anti-diahorrial, hepatoprotective, anti-malarial, antioxidant, anticancer and wound healing properties (10, 11). The major plant-derived chemical groups in *Momordica* species recognized as having potential health promoting effects in diabetes are cucurbitane triterpenoids, saponin glycosides and momordica anti-HIV protein (12). Ramalhete *et al. * (13) have isolated several cucurbitane-type triterpenoids from the aerial parts of *Mormordica balsamina*. Conversely, the multiple medicinal roles of *Leptadenia hastata* have also been documented (14). The leaves and latex of *Leptadenia hastata* have particularly been reported to possess trypanocidal, anti-malarial, antibacterial, anti-inflammatory and sterility properties (15-17). Bello *et al. * (8) documented the hypoglycemic and hypolipidemic effects of the aqueous and methanolic leaf extracts of *Leptadenia hastata* in normal and alloxan-induced diabetic rats. In their study, the aqueous and methanolic extracts of *Leptadenia hastata* reduced blood glucose level and increased liver and muscle glycogen levels.

Furthermore, although many synthetic antidiabetic drugs are available, drugs of natural origin have aroused great interest due to the increased prevalence of diabetes mellitus, side effects and high cost of modern synthetic antidiabetic drugs and availability of plant based medicines (18). The efficacy of many traditional medicinal plants has been confirmed but the active principles of most of these antidiabetic plants used are yet to be characterized. This has prompted an increased scientific research to validate their folkoloric use and bioactive components to develop plant-based drugs that can help to proffer a lasting solution to the burden of diabetes (19). Our previous study demonstrated that the aqueous leaf extracts of *Momordica balsamina* and *Leptadenia hastata* alone and in combination possess significant antihyperglycemic effect in STZ-induced diabetic rats (20). However, the bioactive antidiabetic principles were not characterized. Therefore, in this study, the antidiabetic bioactive principles from the leaves of *Momordica balsamina* and *Leptadenia* *hastata* were isolated and characterized with the application of ^1^H and ^13^C NMR spectroscopy. 

## Experimental

*Materials and methods*

*Plant material and extraction*

The leaves of *Momordica balsamina* and *Leptadenia hastata* were collected in March 2013 from Kano, Nigeria and authenticated by Musa Muhammad at the herbarium of the Department of Biological science, Ahmadu Bello University, Zaria, Nigeria. Voucher specimen for *Momordica balsamina* (1139) and *Leptadenia hastata* (900220) were deposited accordingly for future reference. The collected plant leaves were separated from undesirable plant parts, washed, air-dried and grounded into a fine powder using a mortar and pestle and stored in nylon until required. 

*Extraction and solvent partitioning*

Solvent extraction of the grounded plant leaves was carried out using the Microwave-assisted extraction (MEA) method as described by Ganzler *et al. * (21). The powdered plant leaves (500 grams) moistened with hundred (100) milliliter (mL) of water were successively heated in a microwave oven (Nexus, NX-803) for 3 minutes and cooled to room temperature. The extraction and cooling step was repeated five times and partitioned with solvents (hexane, chloroform, ethylacetate, methanol; 2 × 500 mL each) in ascending order of polarity. Thus, each fraction was filtered with Whatman No 1 filter paper, evaporated to dryness, and acutely screened for antihyperglycemic activity in STZ-induced diabetic rats. The antihyperglycemic screening was carried out with 100 mg/kg b.w of LH and MB crude leaf extracts. Blood samples were collected from rat tail vein and blood glucose level was measured before extract administration at 0 h and at 1, 2, 3, 4, and 6 h post extract administration using a one-touch glucometer (Roche, Germany). 

The ethylacetate and methanolic fractions of MB and LH exhibited the highest percentage decrease in fasting blood glucose, respectively as such were subjected to further isolation and purification using column chromatography and preparative Thin-layer chromatography (PTLC).

*Compounds Isolation and purification*

A column (50 cm^3 ^× 3.5 cm) was packed with n-hexane and silica gel (50 g) using the wet slurry method. The ethyl acetate fraction of MB (4 g) was pre-adsorbed onto silica gel (60–120 mesh, Qualikems) by first solubilizing it in ethyl acetate, followed by the addition of silica gel (1 g) to form a fraction-adsorbent mixture which was dried. The dried fraction - adsorbent mixture (sample) was evenly loaded onto the top of the packed column and eluted with hexane/ethyl acetate (100:0 → 8:2→0:100) as gradient mixture solvent of increasing polarity. Thirty fractions of 100 mL each collected were pooled to afford 6 sub-fractions (M1-M6) based on their thin layer chromatography (TLC) (Silica gel 60F_254_) profile. Fractions M3 and M5 (containing all the compounds in M1-M6) were further separated by preparative thin-layer chromatography (PTLC) to yield compounds H2-H5; H2 and H3 from M3 and H4 and H5 from M5. The quantity of H1 and H6 was not sufficient to carry out any further evaluation.

The methanolic fractions of LH (LHM) were subjected to gel filtration using Sephadex LH-20 gel (5 g, GE Healthcare Biosciences AB), pre swollen in absolute methanol (300 mL). LHM was solubilized in absolute methanol, loaded onto a column (50 cm^3^ × 3.5 cm) and eluted with increasing concentration of methanol up to 100%. Twenty three (23) fractions of 40 mLs each were collected and yielded two sub-fractions (LH1 and LH2) based their TLC profile. A preparative thin-layer chromatography (PTLC) was carried out on the sub-fraction LH2 which yielded 2 compounds; LH2a and LH2b. 

PTLC was performed using silica glass plate 20 × 20 cm and 0.25 mm thickness and the region containing the bands of interest was scraped off, dissolved in methanol and evaporated to obtain pure compounds. The antihyperglycemic activities of the isolated compounds from MBE (H2-H5) and LHM (LH1, LH2a, Lh2b) were evaluated in STZ-induced diabetic rats.

*Antihyperglycemic activity of isolated compounds*

Apparently healthy albino Wistar rats of both sexes having an average weight of 80 ± 2 g were obtained from the animal house of the Department of Zoology, Bayero University, Kano for the experiments. The animals were acclimatized for two weeks before commencing the experiment. The rats were housed in a wire-meshed laboratory cage, fed with commercial pelleted feeds (Vital feeds®, Jos, Nigeria) and watered *ad libitum*. They were kept at 25 ± 2 ºC at natural cycle of light and darkness throughout the experimental period. The experimental procedure was conducted in accordance to the NIH requirements of the guide for care and use of laboratory animals (National Research Council (NRC) (22), and the institutional ethics and research committee of the Faculty of Life Sciences, Ahmadu Bello University, Zaria. 

The experimental animals were made diabetic by a single intraperitoneal injection of freshly prepared solution of streptozotocin (STZ) (60 mg/kg body weight) (Tocoris bioscience, UK) in 0.1 M cold citrate buffer (pH 4.5) after an overnight fast of 12 h (23). The rats were kept for the next 24 h on five (5%) percent glucose solution to prevent STZ induced hypoglycemia (4 h post STZ induction) (24). Diabetes was confirmed by determining fasting blood glucose from blood obtained from the tail vein using a one-touch glucometer (Roche Accu-Check, Germany) seventy two (72) hours post-STZ injection. Rats with a blood glucose level ≥ 250 mg/dL were considered diabetic and selected for this research (25).

*Experimental design*

The experimental animals were randomly assigned into ten groups of three (3) rats in each group and orally treated by gavage with 10 mg/kg body weight in DMSO (26) compounds, drug or vehicle as follows: Group 1: Diabetic + 10 mg/kg MB-H2; Group 2: Diabetic + 10 mg/kg MB-H3; Group 3: Diabetic + 10 mg/kg MB-H4; Group 4: Diabetic + 10 mg/kg of MB-H5; Group 5: Diabetic + 10 mg/kg LH-LH1; Group 6: Diabetic + 10 mg/kg LH-LH2a; Group 7: Diabetic + 10mg/kg LH-LH2b; Group 8: Diabetic + 10 mg/kg glucovance (glibenclamide 2.5 mg/metformin 500 mg) (Merck, Germany); Group 9: Diabetic Control (DMSO); Group 10: Normal control (DMSO). The glucose level of each experimental animal was measured before extract/drug/DMSO administration at 0 h and 1, 2, 4, and 6 h post-administration, using a one-touch glucometer (Roche Accu-Check, Germany). The percentage decrease in fasting blood glucose was calculated for each compound at the end of the experiment. 

*Structural elucidation of antihyperglycemic compounds*

 The identity of the bioactive antihyperglycemic compounds from MBE and LHM were characterized by NMR spectroscopy on Agilent-NMR-vNMR 400MHZ and Bruker Advance Ft-NMR 400MHZ (Bruker BioSpin, Rheinstetten, Germany) (^1^H and ^13^C) NMR spectrometers respectively with TMS as an internal standard. The structure of the compounds was elucidated by comparing their NMR spectral data with literature values. The spatial arrangement of atoms in the compounds was drawn using ACD/Chemsketch (for windows, version 12.0).

*Statistical analysis*

Results were presented as mean ± standard deviation. The Tukey-HSD test was analysed using R statistical software (version 3.2.2, the R foundation for statistical computing platform). Values were considered to be significantly different when *p < *0.05.

## Results

STZ-induction caused a significant (*p < *0.05) increase in blood glucose level when compared to the normal rats (Figures 2 and 3), however, treatment with the crude solvent extracts of *M. balsamina *and *L. hastata* resulted in a significant (*p < *0.05) time-dependent decrease in blood glucose level when compared to the untreated diabetic rats (Figure 2 and 3). The antihyperglycemic activity of the solvent extracts of MB was in the order ethylacetate>methanol>chloroform>hexane while that of LH was in the order methanol>ethylacetate>chloroform>hexane. Thus, the ethylacetate fraction of *M. balsamina* and the methanol fraction of *L. hastata* displayed the highest antihyperglycemic activity at 6 h post extract administration (Table 1). 

The antihyperglycemic activities of the purified fractions from* M. balsamina* and* L. hastata* in STZ-induced diabetic rats revealed that only two fractions were bioactive; H3 from the ethylacetate extract of *M. balsamina* and LH2b from the methanolic extract of *L. hastata* with a percentage decrease in blood glucose of 31.67 ± 6.07% and 34.36 ± 8.17% respectively (Figure 4; Table 2). Although significant antihyperglycemic activities of the two extracts were observed compared to the diabetic controls, the activities were not comparable to the standard drug glucovance^®^.

Compound H3 was isolated as a white compound and was positive for the Liebarmann-Buchard reaction indicating a steroidal nucleus. The ^1^H-NMR (DMSO-d6, 400 MHz) spectral data of H3 (Figure 4) revealed six methyl signals at δ 0.80, 0.81, 0.89, 1.32, 1.10 and at δ 1.54. The ^1^H NMR displayed signals for olefinic hydrogens at δ 4.68, δ 4.58 (2H, s) for H-29 and the hydrogen attached to the carbon bearing the hydroxyl group (H-3) at δ 4.20 (1H, s). The ^13^C-NMR (DMSO-d6, 100 MHz) showed signals for 30 carbon atoms (Figure 4). The peak at δ 170 indicated the presence of a carboxylic group and was assigned to C-28. The signals at δ 150 and 109 were attributed to terminal oleifinic carbons while the peak at δ 79 indicated an oxygenated carbon resonance. These were the diagnostic peaks for pentacyclic triterpenoid of lupane type skeleton and were assigned to C-20, C-29 and C-3 respectively. The ^13^C NMR spectral data (Table 3) were compared with published values (47, 48) and confirmed the identity of H3 as betulinic acid (Figure 5).

The characterized bioactive compound isolated from LHM (LH2b) was found to be 5-methylgenistein; an isoflavonoid. Compound LH2b was isolated as a yellow powder and tested positive for the sodium hydroxide test for flavonoids. The ^1^H NMR (CDCl_3_-Water-DMSO-d6, 400 MHz) spectrum exhibited signals between δ 6 and δ 8 characteristics of splitting of a p-disubstituted benzene derivative, an m-coupled ^1^H signals, and singlets, a pattern similar to that of genistein (49) (Figure 6). The ^13^C NMR (CDCl_3_-Water-DMSO-d6, 100 MHz) exhibited carbonyl signals of a conjugated ketone and an ester group, respectively at δ187 and δ 164. The 6 peaks between δ 161 and δ 148 represented an aromatic carbon atom directly attached to an oxygen atom. A methyl signal at δ 29.5 was exhibited in the aliphatic region. Comparison of spectral data of LH2b (Table 4) with published data of genistein as described by Maskey *et al. * (49) confirmed the identity of LH2b to be 5’-methyl genistein (Figure 7). Comparing the spectral data of our isolated bioactive compound to that of genistein showed negligible difference at the 5’ position of ring B of the isoflavonoid. The peaks revealed an additional methyl group at the 5’ position of the isolated compound.

## Discussion

Triterpenes constitute a large structurally diverse group of natural compounds biogenetically derived from isoprene with common structures such as the pentacyclic- oleanane, ursane, taraxastane, lupane, and tetracyclic-dammarane and cucurbitane (27). Although the presence of a pentacyclic type of triterpenoids has not been documented in *Momordica* species, Zhang *et al. * (28) and Burdi (29) have identified the leaves of *momordica* species as a significant source of triterpenoids. Contrary to our findings, *Momordica* species are shown to contain mainly triterpenoids of the tetracyclic cucurbitane types (28). Thus to the researcher’s knowledge, this is the first report on the successful isolation and identification of a pentacyclic triterpenoid of the lupane type from *Momordica balsamina*. 

Triterpenes especially pentacyclic ones are widely distributed in the plant kingdom and found in the leaves, stem bark, fruits and roots and are frequent objects of phytochemical and pharmacological investigations (30). Many experiments have shown that triterpenes demonstrate several mechanisms of antidiabetic action and are promising agents in the prevention of diabetic complications (31). Numerous *in-vitro *and *in-vivo *studies have revealed their multi-directional biological properties such as the potential to inhibit enzymes involved in glucose metabolism, prevent the development of insulin resistance, normalize plasma glucose levels, and possess hypolipidemic, anti-obesity (32) and antioxidant activities (33). Additionally, prospective studies have reported their ability to inhibit the formation of advanced glycation end products that are implicated in the pathogenesis of diabetic nephropathy, neuropathy, or impaired wound healing (31). Betulinic acid have been reported by several studies to effectively ameliorate hyperglycemia through inhibition of gluconeogenesis (34), elevation of the rates of cellular glucose uptake, inhibition of enzymes involved in glucose metabolism and the ability to normalize plasma glucose and insulin levels (35). Furthermore, the ariel part of *Momordica balsamina* have been shown to be a potent inhibitor of HIV-1 replication *in-vitro *(44). Recent findings suggested that- betulinic acid and its derivatives have anti-HIV, anti-influenza, anti-malarial, anticancer and anti-inflammatory effects (45, 46). Betulinic acid isolated in our study could therefore validate the anti-HIV and antimicrobial activities of* Momordica balsamina*.

The hypoglycemic, hypolipidemic and carbohydrate hydrolysis inhibitory effects of the aqueous and methanol extracts of the leaves of* Leptadenia hastata* in normal, alloxan and streptozotocin induced diabetic rat models have been documented (8, 36). Our preliminary phytochemical screening of the plant did not reveal the presence of flavonoids (50). The presence of a methyl group could have reduced the polarity of methylgenistein, which explains the absence of flavonoids in our preliminary phytochemical screening of the aqueous leaf extract of *Leptadenia hastate.* The number of hydroxyl groups in a compound correlates positively to its radical scavenging activity thus antioxidant effect. Flavonoids, which are ubiquitous components in the plant kingdom, have been shown to decrease blood glucose levels and isoflavonoids are reported to have antioxidant properties, potent inhibition potential to various lytic enzymes as well as antifungal and estrogenic activities (37). 

In addition, many health benefits are attributed to isoflavones and evidence indicates a potential of genistein as a preventative and therapeutic treatment for patients with diabetes mellitus due to its antioxidant potentials. Genistein is documented to be the most abundant isoflavone in nature followed by daidzein. Recent studies have confirmed the antidiabetic effects of genistein particularly its direct effects on β-cell proliferation, glucose-stimulated insulin secretion and protection against apoptosis effects (38). Furthermore, it has been found to manifest hypolipidemic, antioxidant, anti-AGE and anti-obesity activities (31). *In-vitro *studies have documented that genistein inhibits cellular cholesterol synthesis, cholesterol esterification, reduces fatty acid oxidation and exerts lipid lowering effects. Thus, genistein could possess anti-artherogenic activity by acting as anticlotting agent (39). It has been previously investigated for its potential beneficial effects on cancer treatment, cognitive function, and cardiovascular and skeletal health with primary focus on exploring its potential hypolipidemic, antioxidant and esterogenic effects (40-42). Reported pharmaco-medical activities of genistein also include removal of damaging free radicals, reduction of lipid peroxidation and increasing the activity of other antioxidant enzymes such as glutathione peroxidase, superoxide dismutase and glutathione reductase (43).

Future studies on the safety profile of betulinic acid and 5’-methylgenistein to establish their potential therapeutic safe dose range is recommended. Due to the small yield and cost, the study could not evaluate the antidiabetic effects and mechanism of action of the isolated compounds in comparison to known standards. This therefore, is a recommendation for future studies.

**Figure 1:**
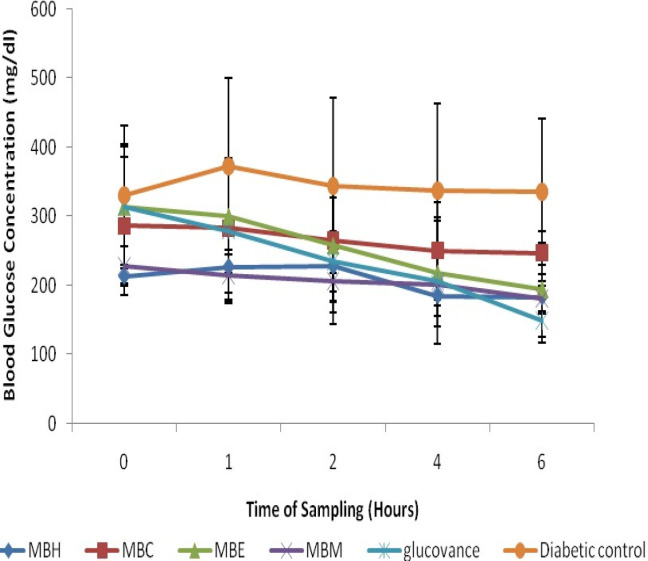
Antihyperglycemic Activities of Hexane, Chloroform, Ethylacetate and Methanolic Fractions of *Mormordica balsamina* in STZ-Induced Diabetic Rats. Values expressed are mean ± SD of three animals for each group; MBH- MB hexane fraction; MBC-MB chloroform fraction; MBE- MB ethylacetate fraction; MBM- MB methanolic fraction

**Figure 2:**
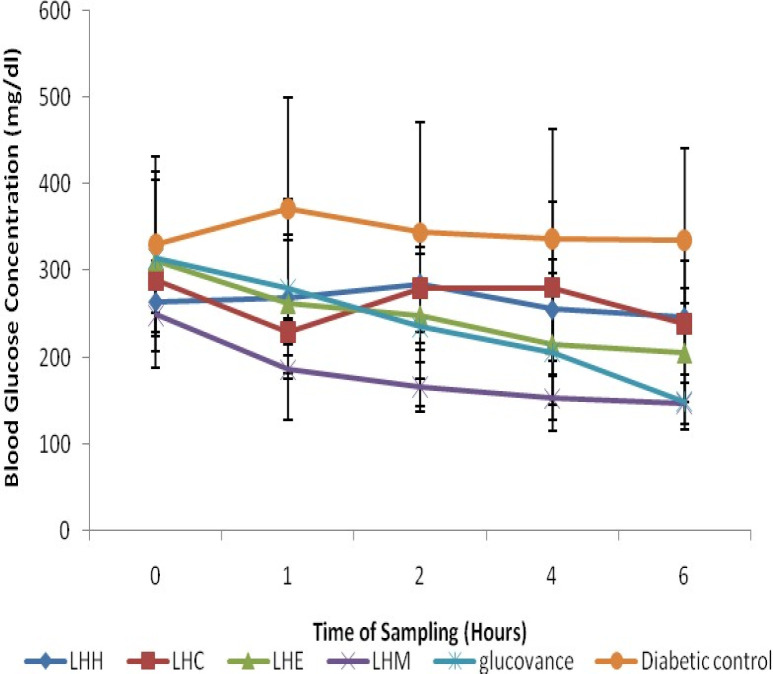
Antihyperglycemic Activities of Hexane, Chloroform, Ethylacetate and Methanolic Fractions of *Leptadenia hastata* in STZ-Induced Diabetic Rats. Values expressed are mean ± SD of three animals for each group; LHH- LH hexane fraction; LHC- LH chloroform fraction; LHE- LH ethylacetate fraction; LHM-LH methanolic fraction

**Figure 3:**
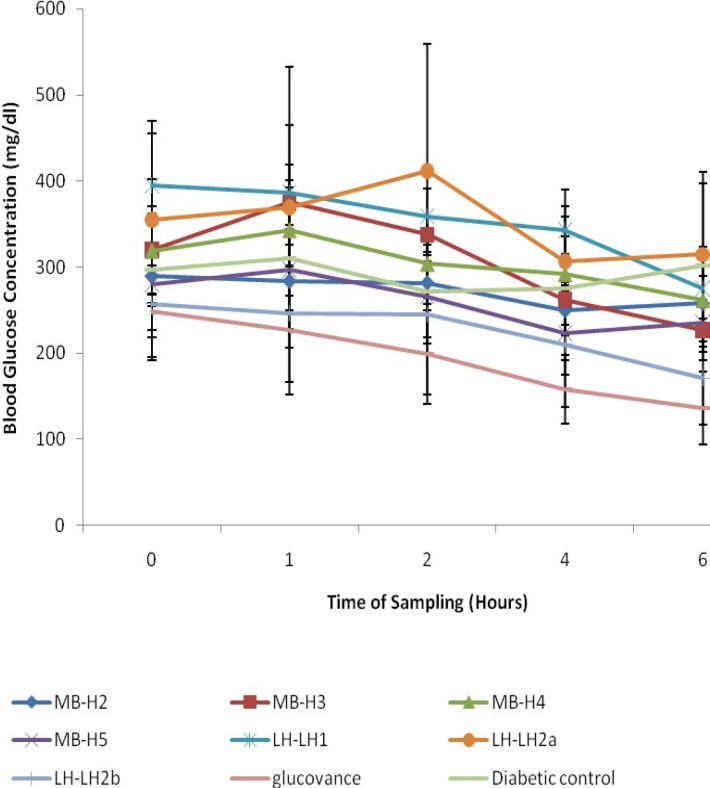
Anti-hyperglycemic Activities of Isolated Compounds of MBEA and LHM

Values expressed are mean ± SD for three animals each.

**Figure 4:**
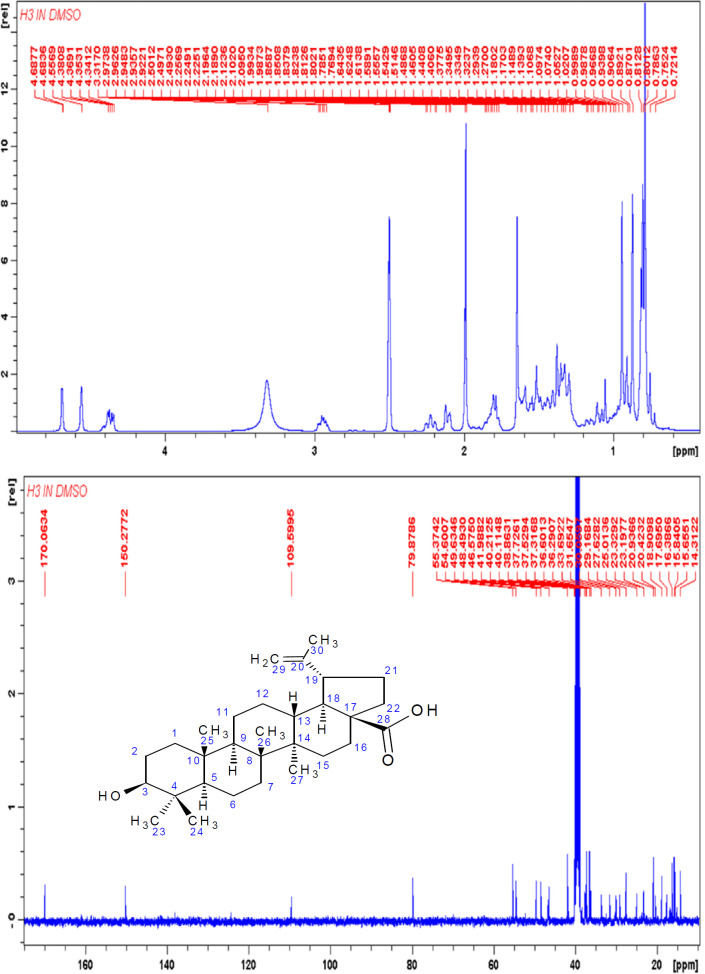
^1^H and ^13^C NMR Spectra of Compound H3

**Figure 5:**
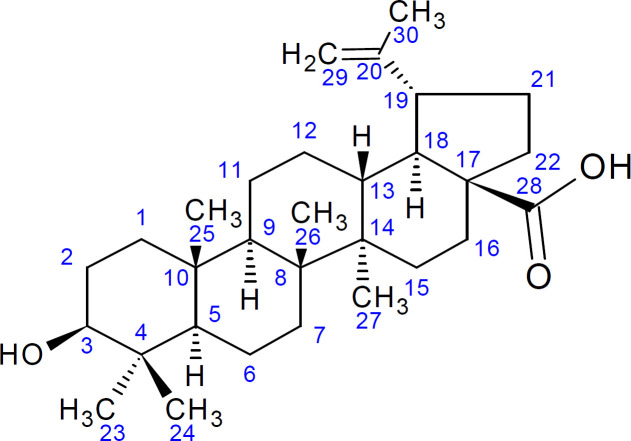
Structure of 3β-hydroxyllup-20(29)-en-28-oic acid (Betulinic acid).

**Figure 6:**
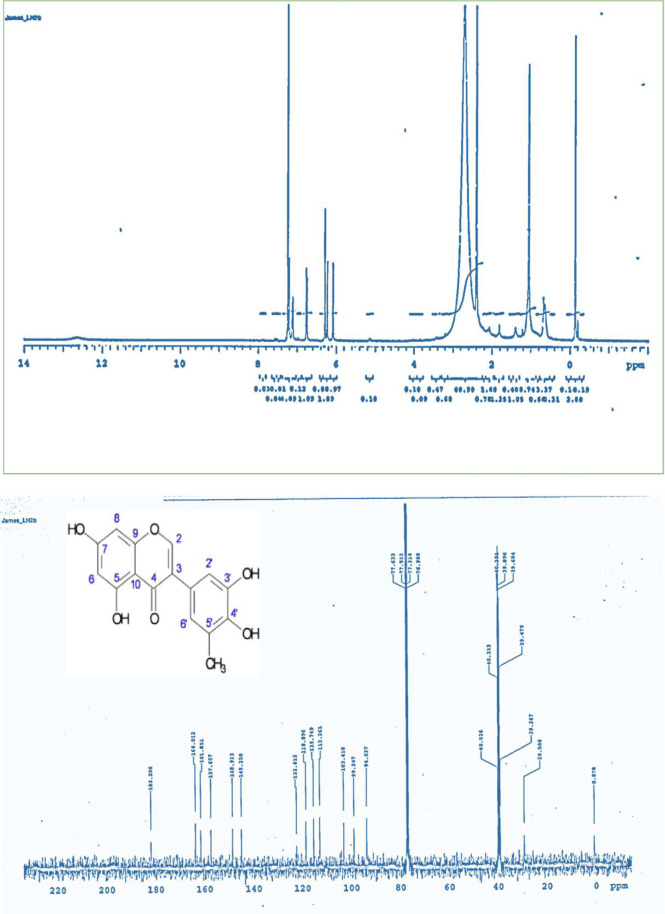
^1^H and ^13^C NMR Spectra of Compound LH2b

**Figure 7:**
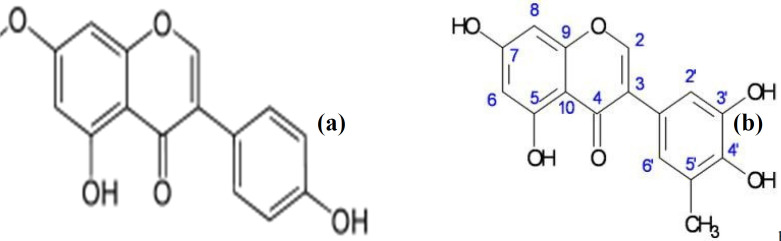
(a) Genistein (b) 5’-MethylGenistein (LH2b)

**Table 1:** Percentage Decrease in Fasting blood glucose (FBG) in STZ-induced diabetic rats treated with the Crude Solvent Fractions of *Leptadenia hastata* and *Momordica balsamina*

**Extracts**	**PERCENTAGE DECREASE in FBG (%)**
MBH	0.21 ± 7.81
MBC	2.09 ± 3.66
MBE	37.93 ± 14.28
MBM	20.52 ± 4.93
LHH	8.52 ± 12.34
LHC	17.52 ± 5.04
LHEA	32.25 ± 12.70
LHM	40.32 ± 4.87
Normal Control	0.96 ± 3.84
Diabetic Control	4.62 ± 8.17

Results expressed are mean ± SD of three animals for each group; FBS- fasting blood glucoae; MBH- MB

hexane fraction; MBC-MB chloroform fraction; MBE- MB ethylacetate fraction; MBM- MB methanolic fraction;

LHH- LH hexane fraction; LHC- LH chloroform fraction; LHE- LH ethylacetate fraction; LHM-LH methanolic fraction.

**Table 2:** Antihyperglycemic Activities of Isolated Compounds of MBE and LHM

**Compounds**	**PERCENTAGE DECREASE IN FBG (%)**
MB-H2	^b^10.45 ± 3.23
MB-H3	^ab^31.67 ± 6.01
MB-H4	^b^17.28 ± 6.10
MB-H5	^b^16.11 ± 3.04
LH-LH1	^ab^30.12 ± 6.61
LH-LH2a	^b^11.24 ± 2.06
LH-LH2b	^ab^34.36 ± 8.17
Glucovance	^a^45.96 ± 12.51
Diabetic Control	4.62 ± 8.18

Results are expressed as mean ± SD *p < *0.05 ^a^ (Comparison with Diabetic Control) _b_ (Comparison with Diabetic

Glucovance). MB (H2-H5)- Isolated compounds of Ethylacetate fraction of MB; LH (LH1-LH2a-b)- Isolated

compounds of Methanolic fraction of LH.

**Table 3:** Comparison of Chemical Shifts of Compound H3 in DMSO-d6 with reported values of betulinic acid

**Carbon Position **	^13^ **C Shift (ppm)** **Experimental**	^13^ **C Shift (ppm) Lit Value**	**CH**_n_
1	38.66	38.70	CH_2_
2	27.26	27.40	CH_2_
3	79.58	78.90	CH
4	38.76	38.80	C
5	55.26	55.30	CH
6	18.20	18.30	CH_2_
7	34.25	34.30	CH_2_
8	40.62	40.70	C
9	50.42	50.50	CH
10	37.06	37.20	C
11	20.78	20.80	CH_2_
12	25.43	25.50	CH_2_
13	38.10	38.80	CH
14	42.32	42.40	C
15	30.56	30.50	CH_2_
16	32.22	32.10	CH
17	55.99	56.30	C
18	46.80	46.80	CH_2_
19	49.09	49.20	CH_2_
20	150.27	150.30	C
21	29.61	29.70	CH_2_
22	37.06	37.00	CH_2_
23	27.67	27.90	CH_3_
24	15.42	15.30	CH_3_
25	15.94	16.00	CH_3_
26	16.04	16.10	CH_3_
27	14.58	14.70	CH_3_
28	170.06	178.43	COOH
29	109.60	109.60	CH_2_
30	19.27	19.40	CH_3_

Lit ^13^C Values (Habila *et al. ,* 2010; Mahato and Kundu, 1994).

**Table 4:** Comparison of Chemical Shifts of Compound LH2b in CDCl_3_-Water-DMSO-d6 with Reported Values of Genistein

**Carbon Position **	^13^ **C Shift (ppm)** **Experimental**	^13^ **C Shift (ppm) Lit Value**	**CH**_n_
1	-	-	-
2	157.7	153.05	CH_2_
3	122.6	126.52	CH
4	187.2	180.86	C
5	164.0	164.81	CH
6	99.2	99.87	CH_2_
7	164.0	164.21	CH_2_
8	94.1	94.47	C
9	161.9	162.50	CH
10	103.4	105.8	C
1’	122.6	127.48	CH_2_
2’	113.3	114.73	CH_2_
3’	145.3	141.54	CH-OH
4’	148.9	147.5	C
5’	118.9	124.03	CH_2_
6’	115.7	120.3	CH
CH_3 _	29.5*	21.75	CH_3_

^13^C Lit Values Maskey *et al.* (2003).

## Conclusion

Our study on *M. balsamina* and *L. hastata* led to the isolation of betulinic acid and 5’-methylgenistein as one of their potential bioactive antidiabetic constituents respectively. This further validates the antihyperglycemic effect of the polyherbal combination, thus its use to manage diabetes mellitus and its associated complications. 
